# On the Inhalation of Etherial Vapor for the Prevention of Pain during Surgical Operations

**Published:** 1847-02

**Authors:** 


### PART IV.—EDITORIALS.

ARTICLE XIII.

### ON THE INHALATION OF ETHERIAL VAPOR FOR THE PREVENTION OF PAIN DURING SURGICAL OPERATIONS.

Every one has heard before this of the use of the "narcotic vapour," or "Lethion," for the prevention of pain during operations. As usual in similar cases, a number of claimants have arisen to contest the honor of the discovery. The credit seems to rest between Dr. Charles T. Jackson, a chemist of Boston, and Dr. Morton, a dentist of that city, who are named in the patent as inventors, the latter gentleman being proprietor,* and Horace Wells, a dentist of Hartford, who went to Boston, proposed its use to Drs. Morton and Jackson among others, but not succeeding well in its first trials, gave it up for want of encouragement. Mr. Wells, however, preferred the nitrous oxide to the ether, thinking it to be less dangerous. Drs. Jackson and Morton patented the ether, thinking it more convenient probably, or perhaps finding its effects more favorable.† The facts, if correctly stated, seem fully to vindicate the claim of Mr. Wells. So much for the discoverer.

*Composition.*—In regard to the substance made use of, its composition has not been made public by the patentees, but it is used everywhere with and without their permission, and is nothing else but pure rectified sulphuric ether. This at least has every effect ascribed to the patent vapor, although various preparations, consisting of sulphuric and chloric ethers with morphine, in form of sulphate or acetate, in different proportions, are in use. Sometimes a small quantity of etherial oil is added, but the addition of any or all these substances, seems neither to add to, or detract from, the effect of the sul ether.

*Method of using it.*—For this purpose a receiver is employed, with two orifices, as a two-necked bottle, to one of the orifices of which is attached a tube, furnished at its extremity with a mouth piece like that of a speaking trumpet, to fit the lips. In this tube is a valve, opening toward the mouth, so as

* Boston Med. & Surg. Journal for Nov. 18, 1846, p. 316.
† Ib. of Dec. 16. p. 398.

to allow the vapor to be inhaled, and closing to prevent it from being forced again into the bottle. When expired it escapes by an orifice in the side of the tube, which closes with a valve during inspiration. The receiver should be sufficiently large to hold a pint and filled with a sponge to present a large evaporating surface. An inhaling apparatus may be procured in Chicago for one dollar. As much ether should be put in the receiver as the sponge will absorb, and the orifices may be closed with corks until it is required for use. The patient being seated, if convenient, upon an easy chair, (or a recumbent position if required will do,) the nostrils should be closed and the mouth piece applied to the lips in such a manner as to prevent the introduction of air. The patient being directed to inhale and fill the lungs freely.

*Effects on the System.*—The first contact of the vapor with the air passages is often attended by a slight cough. After a few inhalations there are frequently manifest signs of excitement, such as clenching the fist, or efforts to rise, which, if restrained, immediately cease, and the patient falls into a state of profound insensibility. The respiration is slow and slight, the pulse is frequently reduced, at first in frequency, afterwards accelerated, the pupils are sometimes dilated, at other times natural, the person has all the appearances of being in a state of profound sleep.

These effects continue only a few minutes if the inhalation is suspended as soon as they are noticed, but if continued longer, they are more permanent; and there are a few cases on record in which the insensibility continued an hour or more.

The length of time required for inducing sleep varies in different cases according to the quantity of the vapor mingled with the air, and according also to the susceptibility of the system; we have seen very perfect sleep induced in one and a half minutes, in other cases it required seven or eight minutes, but there was a possibility of the vapor being weaker in these latter trials. During the inhalation a physician should feel the pulse, notice the effect on the respiration, observe whether the face is flushed or the veins of the neck distended. If any unusual effects are observed suspend the inhalation altogether, if not, continue till the sleep is perfect then discontinue, to renew it if signs of returning sensibility should appear before the operation is finished. It often happens that the patient will move and speak, as in somnambulism, and yet be insensible to pain, although at the moment of being hurt he may give signs of suffering. A young lady cried out when a tooth was extracted, but on recovery was unconscious of having suffered at all, but said she had a dream, and thought she was dreaming. These effects we have described from our own observations, and they correspond to those noticed by other persons in this city, upon probably from one to two hundred individuals to whom it has, up to the present time, been administered. But in a report signed by "twelve regular dentists," of Boston,* the common or frequent use of the article in dentistry is deprecated, on account of accidents which are stated to have been produced by its use. These effects were—1st, the long continuance of the state of insensibility; 2nd, the occurrence of delirium; 3d, the occurrence of nervous symptoms, as weeping, sighing, &c.; 4th, in one instance the patient "raised blood from the lungs, about a pint, and was suffering in consequence of the operation for three days after." From which this committee of regular dentists very justly conclude, "that this whole matter, be it of greater or less utility in surgical practice, should be put in the hands of those only who have testimonials from some one of our medical colleges, that they are worthy of being entrusted, as medical attendants, with the health and life of their fellow beings."

The exact nature of the effect resulting from the administration of the ether has been the subject of speculation; some supposing it to be a species of asphyxia, others that it results from temporary congestion of the brain, and others still, that it is a species of intoxication. It appears to bear no resemblance to asphyxia, the breathing being gentle and the veins of the neck being not at all turgid, showing that there is no obstacle to the circulation. Neither does it resemble congestion or depression of the brain, as is shown by the dreams, and absence of stutorous breathing, and fullness of the vessels attendant on congestion. The resemblance to intoxication is greater but not by any means perfect.

It seems to be a state of sleep or trance in which the sensibility is blunted, but the activity of certain of the mental faculties preserved.

* Ib. for Jan. 6, 1847, p. 472.

*Uses.*—It has thus far been used principally for preventing pain during the operations of extracting teeth and of amputating members, although there are some cases on record in which it has been employed before and during the extirpation of tumors, &c. A surgeon of the Massachusetts Hospital used it for the purpose of producing relaxation of the muscles while reducing a dislocation of the shoulder. It has however been proposed to use it for favoring the operation of the taxis in strangulated hernia, and it would be worthy of trial in a great number of severe neuralgic and spasmodic diseases.

Case 1.—On the 12th of January, 1847, a young man presented himself at the dispensary of Rush Medical College for amputation of the middle finger, rendered necessary by necrosis of the first phalanx, of four months standing. Being very timid, he wished something to prevent the pain, and the etherial vapor was administered to him for about five minutes, in a moderately concentrated form, when he became insensible. The finger and the head of the metacarpal bone were removed. He remained asleep a minute after the operation was finished, and on awaking and perceiving that it was done he smiled, and said, "that was pretty easy." No ill effects followed; he remained about a week in the city and returned to his home, on Fox River.

Case 2.—On the 24th inst., a young gentleman, also from Fox River, came to the dispensary, for the purpose of having some of the metatursal bones removed, for a caries and necrosis of long standing. The ether was inhaled in a concentrated form, signs of excitement were at first shown, but in a minute and a half he became insensible. The disease proving more extensive than appearances indicated, it became necessary to remove the metatursal bones of the three lesser toes, which were soft, and in fragments in the tissues. The operation was not completed until twenty-five minutes had elapsed, during which time the vapor had been inhaled five times; two or three inhalations being given as soon as signs of returning consciousness was perceived. Considerable blood was lost and partial syncope induced. On recovering he stated that he had scarcely any recollection of pain. No disagreeable consequences followed and he is doing well. The parts divided were in a state of inflammation, and few operations are more painful or protracted. So that the test of the powers of ether was, in this instance, very perfect.

Desirous of knowing with greater certainty the effects resulting from long continuance of the operation, a large dog was made to inhale the vapor, in a concentrated form, for eight minutes. After a few inhalations he became insensible, stuggled feebly from time to time, and remained in a state of relaxation for a few seconds, with his head hanging from the table, when he recovered entirely. This experiment without being altogether satisfactory, goes far to show that the respiration of the vapor, even when concentrated and used sufficiently long to produce its full effects on the animal system, is not likely to be directly followed by any effects dangerous to life; but a series of experiments upon the lower animals is a desideratum, and until they have been performed we shall be in ignorance of the effects it is liable to occasion if prolonged much beyond the period necessary for producing insensibility. We have not time at present to prosecute these researches, but may do so at some future time unless the void should soon be filled by others.

At present we are justified in the conclusion, that with the precautions above recommended, this vapor may be generally administered with safety. The exceptions to this rule are those cases where there is disease of the lungs, tendency to apoplexy, or mania, or an extreme nervous temperament. In children too it is said to produce vomiting, and it is besides, in such cases, difficult to make them respire it.

Is the discovery the subject for a patent? The Boston committee of dentists state that they have consulted high legal authority, who are of opinion that the patent is not valid. We have also consulted several legal gentlemen of high standing in Illinois who are of the same opinion; but as the question will soon be brought before the proper tribunals for adjudication, it is not necessary to discuss it at present. Another, on which there will be less difference of opinion among scientific men, is in regard to the propriety of a physician taking out a patent for any improvement in medicine. This is contrary to any correct principle of medical ethics, and whatever may be said of preventing its abuse, and the liberal intentions of the patentees in regard to granting the right to use it to surgeons, it still presents them in the same attitude as the proprietors of patent medicines; in great and strong contrast, with those honorable and benevolent physicians who are above keeping secret what may be useful to their fellow men, and who are rejoiced to see the discoveries they may make widely adopted, and universally beneficial.

As to the ultimate decision of the world upon the value of this means of preventing pain, it is impossible to foresee what it will be. It has already found advocates and opponents whose zeal is little calculated to favor the discovery of its true value. Some pronounce it free from danger, others think it unworthy of a trial. It has seemed to us to demand the most careful and dispassionate examination of surgeons.

We have given the results of our own observations, and add elsewhere such facts and opinions, from other sources, as will give our readers some idea of the great interest which it excites throughout the country.

